# The Causal Effect of Vitamin D Binding Protein (DBP) Levels on Calcemic and Cardiometabolic Diseases: A Mendelian Randomization Study

**DOI:** 10.1371/journal.pmed.1001751

**Published:** 2014-10-28

**Authors:** Aaron Leong, Waheed Rehman, Zari Dastani, Celia Greenwood, Nicholas Timpson, Lisa Langsetmo, Claudie Berger, METASTROKE, Lei Fu, Betty Y. L. Wong, Suneil Malik, Rainer Malik, David A. Hanley, David E. C. Cole, David Goltzman, J. Brent Richards

**Affiliations:** 1 Centre for Clinical Epidemiology, Lady Davis Institute for Medical Research, Jewish General Hospital, McGill University, Montreal, Quebec, Canada; 2 Department of Medicine, McGill University, Montreal, Quebec, Canada; 3 Segal Cancer Centre, Lady Davis Institute for Medical Research, Jewish General Hospital, McGill University, Montreal, Quebec, Canada; 4 Department of Oncology, McGill University, Montreal, Quebec, Canada; 5 Department of Epidemiology, Biostatistics and Occupational Health, McGill University, Montreal, Quebec, Canada; 6 Department of Human Genetics, McGill University, Montreal, Quebec, Canada; 7 MRC Integrative Epidemiology Unit, University of Bristol, Bristol, United Kingdom; 8 CaMos Coordinating Centre, McGill University, Montreal, Quebec, Canada; 9 Department of Laboratory Medicine and Pathobiology, University of Toronto, Toronto, Ontario, Canada; 10 Department of Clinical Pathology, Sunnybrook Health Sciences Centre, Toronto, Ontario, Canada; 11 Office of Biotechnology, Genomics and Population Health, Public Health Agency of Canada, Toronto, Ontario, Canada; 12 Institute for Stroke and Dementia Research, Klinikum der Universität München, Ludwig-Maximilians-Universität, Munich, Germany; 13 Division of Endocrinology and Medicine, Department of Medicine, University of Calgary, Calgary, Alberta, Canada; 14 Department of Twin Research and Genetic Epidemiology, King's College London, London, United Kingdom; Imperial College London, United Kingdom

## Abstract

**Background:**

Observational studies have shown that vitamin D binding protein (DBP) levels, a key determinant of 25-hydroxy-vitamin D (25OHD) levels, and 25OHD levels themselves both associate with risk of disease. If 25OHD levels have a causal influence on disease, and DBP lies in this causal pathway, then DBP levels should likewise be causally associated with disease. We undertook a Mendelian randomization study to determine whether DBP levels have causal effects on common calcemic and cardiometabolic disease.

**Methods and Findings:**

We measured DBP and 25OHD levels in 2,254 individuals, followed for up to 10 y, in the Canadian Multicentre Osteoporosis Study (CaMos). Using the single nucleotide polymorphism rs2282679 as an instrumental variable, we applied Mendelian randomization methods to determine the causal effect of DBP on calcemic (osteoporosis and hyperparathyroidism) and cardiometabolic diseases (hypertension, type 2 diabetes, coronary artery disease, and stroke) and related traits, first in CaMos and then in large-scale genome-wide association study consortia. The effect allele was associated with an age- and sex-adjusted decrease in DBP level of 27.4 mg/l (95% CI 24.7, 30.0; *n* = 2,254). DBP had a strong observational and causal association with 25OHD levels (*p* = 3.2×10^−19^). While DBP levels were observationally associated with calcium and body mass index (BMI), these associations were not supported by causal analyses. Despite well-powered sample sizes from consortia, there were no associations of rs2282679 with any other traits and diseases: fasting glucose (0.00 mmol/l [95% CI −0.01, 0.01]; *p* = 1.00; *n* = 46,186); fasting insulin (0.01 pmol/l [95% CI −0.00, 0.01,]; *p* = 0.22; *n* = 46,186); BMI (0.00 kg/m^2^ [95% CI −0.01, 0.01]; *p* = 0.80; *n* = 127,587); bone mineral density (0.01 g/cm^2^ [95% CI −0.01, 0.03]; *p* = 0.36; *n* = 32,961); mean arterial pressure (−0.06 mm Hg [95% CI −0.19, 0.07]); *p* = 0.36; *n* = 28,775); ischemic stroke (odds ratio [OR] = 1.00 [95% CI 0.97, 1.04]; *p* = 0.92; *n* = 12,389/62,004 cases/controls); coronary artery disease (OR = 1.02 [95% CI 0.99, 1.05]; *p* = 0.31; *n* = 22,233/64,762); or type 2 diabetes (OR = 1.01 [95% CI 0.97, 1.05]; *p* = 0.76; *n* = 9,580/53,810).

**Conclusions:**

DBP has no demonstrable causal effect on any of the diseases or traits investigated here, except 25OHD levels. It remains to be determined whether 25OHD has a causal effect on these outcomes independent of DBP.

*Please see later in the article for the Editors' Summary*

## Abstract 2

In this study, Richards and colleagues undertook a Mendelian randomization study to determine whether vitamin D binding protein (DBP) levels have a causal effect on common calcemic and cardiometabolic diseases. They concluded that DBP has no demonstrable causal effect on any of the diseases or traits investigated here, except Vit D levels.

*Please see later in the article for the Editors' Summary*

## Editors' Summary

**Background:**

Vitamin D deficiency is an increasingly common public health concern. According to some estimates, more than a billion people worldwide may be vitamin D deficient. Indeed, many people living in the US and Europe (in particular, elderly people, breastfed infants, people with dark skin, and obese individuals) have serum (circulating) 25-hydroxy-vitamin D (25OHD) levels below 50 nmol/l, the threshold for vitamin D deficiency. Vitamin D helps the body absorb calcium, a mineral that is essential for healthy bones. Consequently, vitamin D deficiency can lead to calcemic diseases such as rickets (a condition that affects bone development in children), osteomalacia (soft bones in adults), and osteoporosis (a condition in which the bones weaken and become susceptible to fracture). We get most of our vitamin D needs from our skin, which makes vitamin D after exposure to sunlight. Vitamin D is also found naturally in oily fish and eggs, and is added to some other foods, including cereals and milk, but some people need to take vitamin D supplements to avoid vitamin D deficiency.

**Why Was This Study Done?:**

Observational studies have reported that the low levels of serum 25OHD and serum vitamin D binding protein (DBP, a key determinant of serum 25OHD level) are both associated with the risk of several common diseases and traits. Such studies have implicated vitamin D deficiency in cardiometabolic disease (cardiovascular diseases that affect the heart and/or blood vessels and metabolic diseases that affect the cellular chemical reactions needed to sustain life), in some cancers, and in Alzheimer disease. But observational studies cannot prove that vitamin D deficiency or DBP levels actually cause any of these diseases. So, for example, an observational study might report an association between vitamin D deficiency and type 2 diabetes (a metabolic disease), but the individuals who develop type 2 diabetes might share another unknown characteristic that is actually responsible for disease development (a confounding factor). Alternatively, type 2 diabetes might reduce circulating vitamin D levels (reverse causation). Here, the researchers undertake a Mendelian randomization study to determine whether circulating DBP levels have causal effects on calcemic and cardiometabolic diseases. In Mendelian randomization, causality is inferred from associations between genetic variants that mimic the influence of a modifiable environmental exposure and the outcome of interest. Because gene variants are inherited randomly, they are not prone to confounding and are free from reverse causation. So, if low DBP levels lead to low serum 25OHD levels, and vitamin D levels have a causal effect on common diseases, genetic variants associated with low DBP levels should be associated with the development of common diseases.

**What Did the Researchers Do and Find?:**

The researchers analyzed the association between a genetic variant called single nucleotide polymorphism (SNP) rs2282679, which is known to alter DBP levels, and calcemic and cardiometabolic diseases and related traits in 2,254 participants in the Canadian Multicentre Osteoporosis Study (CaMos). The researchers report that there was a strong association between SNP rs2282679 and both serum DBP and 25OHD levels among the CaMos participants. However, there were no significant associations (associations unlikely to have occurred by chance) between SNP rs2282679 and calcium level, osteoporosis, or several cardiometabolic diseases, including heart attacks and diabetes. Moreover, when the researchers examined publically available genome-wide association study data collected by several international consortia investigating genetic influences on disease, they found no significant associations between rs2282679 and a wide range of calcemic and cardiometabolic diseases.

**What Do These Findings Mean?:**

In this Mendelian randomization study, DBP level had no demonstrable causal effect on any of the calcemic or cardiometabolic diseases or traits investigated, except 25OHD level. Because most of the participants in CaMos and the international consortia were of European descent, these findings are applicable only to people of European ancestry. Moreover, like all Mendelian randomization studies, the reliability of these findings depends on several assumptions made by the researchers. Notably, although this study strongly suggests that DBP level does not have a causal influence on several common diseases, it remains to be determined whether 25OHD has a causal effect on any calcemic or cardiometabolic outcomes independent of DBP level.

**Additional Information:**

Please access these websites via the online version of this summary at http://dx.doi.org/10.1371/journal.pmed.1001751.

- The UK National Health Service Choices website provides information about vitamin D and about how to get vitamin D from sunshine; “Behind the Headlines” articles describe a recent observational study that reported an association between vitamin D deficiency and Alzheimer disease and the media coverage of this study, other health claims made for vitamin D, and a randomized control trial that questioned the role of vitamin D in disease
- The US National Institutes of Health Office of Dietary Supplements provides information about vitamin D (in English and Spanish)
- The US Centers for Disease Control and Prevention provides information about the vitamin D status of the US population
- MedlinePlus has links to further information about vitamin D (in English and Spanish)
- Information about the Canadian Multicentre Osteoporosis Study is available
- Wikipedia has a page on Mendelian randomization (note: Wikipedia is a free online encyclopedia that anyone can edit; available in several languages)

## Introduction

Serum vitamin D levels are generally low in populations worldwide [1],[2]. Observational studies have implicated vitamin-D-deficient states in several common diseases and related traits, including cardiovascular disease [3]–[5], type 2 diabetes [6],[7], breast cancer [8], bladder cancer [9], colorectal cancer [10], chronic lung diseases such as asthma, bronchiectasis, and other lung infections [11]–[14]. Vitamin D may have modulatory effects on the immune system and thus a potential effect on rheumatologic, inflammatory, and autoimmune diseases [15],[16], such as type 1 diabetes, inflammatory bowel disease [17], familial Mediterranean fever [18], multiple sclerosis [19], and chronic pain [20],[21].

Although observational studies have shown associations between vitamin D deficiency (defined as serum 25-hydroxy-vitamin D [25OHD] level <50 nmol/l) and a number of common diseases, it is not known whether these relationships are causal. Similarly, while animal studies have provided some mechanistic clues into possible causal pathways, mechanisms underlying the associations between circulating 25OHD concentrations and different diseases remain unclear. One key determinant of 25OHD levels is vitamin D binding protein (DBP), a group-specific component of serum globulin [22]. DBP acts as the principal protein carrier for serum 25OHD and activated vitamin D, and thus functions as a reservoir for vitamin D metabolites [23]. Approximately 85%–90% of 25OHD is transported from the liver to target organs bound to DBP [24]. DBP also affects the clearance of vitamin D metabolites in part by aiding in the reabsorption of filtered vitamin D metabolites through megalin in the kidney [25].

DBP is encoded by *GC*, which is located on Chromosome 4q13. Genetic association testing of a single nucleotide polymorphism (SNP) at *GC*, rs2282679, in the TwinsUK cohort (*n* = 1,674) showed that this genetic variant was strongly associated with DBP concentrations (*p* = 4.0×10^−42^) [26]. The Study of Underlying Genetic Determinants of Vitamin D and Highly Related Traits (SUNLIGHT), a large, multicenter genome-wide association study (GWAS), demonstrated that rs2282679 was also strongly associated with reduced 25OHD concentrations (*p* = 1.9×10^−109^) [26]. Each copy of the effect allele, C, at rs2282579, lowered 25OHD levels by an amount similar to that associated with daily vitamin D supplementation [26]. Other genetic studies have also shown robust associations between *GC* genetic variants and circulating 25OHD concentrations [26]–[29].

If vitamin D has a causal impact on common diseases and DBP levels are a main determinant of 25OHD levels, DBP may lie in the causal pathway between 25OHD and these conditions. Indeed, DBP may have additional metabolic roles beyond vitamin D transport. For example, it may modulate bone development, innate immunity, and inflammatory responses [30]–[32]. DBP has also been purportedly linked with autoimmune and inflammatory diseases, aspirin resistance (i.e., failure of aspirin to inhibit platelet function) [33], various arthritides [34], and cardiometabolic outcomes [35]–[37]. Polymorphisms in the *GC* gene [38] have been associated with oral glucose tolerance, fasting insulin levels [39], fracture risk [40], and breast cancer [41]. Thus, observational evidence showing that differences in serum DBP levels are associated with common diseases supports the hypothesis that DBP may either be an intermediate in the biological pathway or an upstream determinant influencing 25OHD effects on common diseases. By way of analogy, it has been suggested that other transporters of steroidal hormones, including sex hormone binding globulin and corticosteroid binding globulin, are major effectors of steroid action independent of their function as carriers [42].

We therefore examined whether DBP levels might be associated with common diseases, potentially clarifying some of the mechanisms underpinning the possible clinical effects of 25OHD. Mendelian randomization (MR) is a method used to provide evidence for *causal* associations between a potentially modifiable risk factor (e.g., DBP) and common diseases when a randomized clinical trial is not possible [43]. The MR paradigm relies on the random assortment of genetic variants at conception to provide an un-confounded study design to estimate the *causal* effects of an intermediate phenotype on an outcome measure of interest (i.e., disease and related phenotypic traits) undistorted by reverse causation [43]. Findings from such an analysis can strengthen causal inferences from observational studies and could therefore clarify whether DBP is in the causal pathway between 25OHD levels and disease.

Here, we first evaluated the causal impact of DBP levels on (1) serum 25OHD concentrations, (2) 25OHD biological readouts, such as parathyroid hormone (PTH) levels, and (3) common cardiometabolic diseases that have been related to vitamin D physiology and their associated traits, through a MR analysis comprising two stages. In the first stage, we determined the effect of DBP levels on these diseases and traits in a large, multicenter cohort. In the second stage, we sought replication of findings in large-scale international GWAS consortia, to achieve a maximal sample size and precise effect estimates. We tested whether these causal estimates from MR analyses were consistent with findings from observational analyses.

## Methods

### Study Populations

The Canadian Multicentre Osteoporosis Study (CaMos) population was used to describe the relationship between rs2282679, 25OHD, and DBP levels, and to produce instrumental variable (IV) results on the causal effect of DBP levels on diseases and related traits. CaMos is an ongoing population-based prospective cohort study of 9,423 randomly selected community-dwelling women (*n* = 6,539) and men (*n* = 2,884) aged ≥25 y at baseline (1995–1997) residing in Canada [44]. Interviews and measurements were repeated at 5 and 10 y after enrollment; participant retention remained at 67% at the tenth year. A similar approach was used to enroll a youth cohort of individuals aged 16–24 y at recruitment (2004–2006). Ethics approval for the study was obtained from the ethics review board at each institution involved in the study, and all participants gave written informed consent in accordance with the Helsinki declaration. The current study sample included 2,254 participants ≥16 y of age for whom genotyping for SNP rs2282679 was obtained (adult cohort, *n* = 2,122; youth cohort, *n* = 132).

The large-scale GWAS international consortia used to replicate CaMos findings of the influence of rs2282679 on traits and disease were the following: (1) METASTROKE, a collaborative meta-analysis of ischemic stroke GWAS data comprising 15 cohorts (12,389 cases/62,004 controls) of European ancestry from the International Stroke Genetics Consortium (ISGC) [45]; (2) the Genetic Factors for Osteoporosis Consortium (GEFOS), which meta-analyzed genetic data and bone mineral density (BMD) measurements from 17 GWASs comprising 32,961 individuals of European and East Asian ancestry [46]; (3) the Diabetes Genetics Replication and Meta-analysis (DIAGRAM) Consortium, which combined samples from 9,580 cases of type 2 diabetes and 53,810 controls of European ancestry [47]; (4) the International Consortium for Blood Pressure (ICBP), which meta-analyzed genetic data on blood pressure indices from 28,775 individuals of European descent [48]; (5) the Meta-analyses of Glucose and Insulin-Related Traits Consortium (MAGIC), which meta-analyzed 21 GWASs comprising up to 46,186 non-diabetic individuals to identify genetic loci that impact glycemic and metabolic traits [49]; (6) the Genetic Investigation of Anthropometric Traits (GIANT) Consortium, which meta-analyzed data from ∼170,000 participants to identify genetic loci associated with body mass index (BMI) and other anthropometric traits [50]; (7) the Coronary Artery Disease Genome Wide Replication and Meta-analysis (CARDIoGRAM), a meta-analysis of 14 GWASs of individuals of European descent for coronary artery disease [51]; and (8) SUNLIGHT, a meta-analysis GWAS of 33,996 individuals from 15 cohorts in Europe and North America [26].

### CaMos Data Collection

Data collection at baseline employed an extensive interviewer-administered questionnaire that included socio-demographic information, medical and family history, general health, medication and supplement use, and food intake. “Ethnicity” was determined by self-report; indication of an ethnicity other than “white” or “European” was considered “non-European.” Disease status was ascertained with the following survey question: “Have you ever been told by a doctor that you have any of the following conditions: hypertension, insulin-dependent or non-insulin-dependent diabetes, heart attack, osteoporosis, stroke, or transient ischemic attack, among other health conditions?” Participants were considered a positive case if they had ever responded “yes” during one of the interviews conducted in year 0, 5, or 10. We investigated cardiometabolic and calcium-related common diseases with a prevalence of at least 5% (*n*>100) in the study population.

### Clinical Assessments

Clinical assessments included height and weight measurements to calculate BMI using the following formula: weight (in kilograms)/height (in meters)^2^. BMD was measured at the femoral neck using a DXA Hologic QDR 1000, 2000, or 4500 bone densitometer (Hologic, Marlborough, Masschusetts, US) or a Lunar DPX bone densitometer (Lunar, Piscataway, New Jersey, US). BMD at the femoral neck was chosen as the standard osteoporosis-related outcome as it is not confounded by non-osteoporotic degenerative changes. Lunar data were converted into equivalent Hologic values by standard methods [52],[53]. A detailed description of BMD quality control appears elsewhere [54].

### Laboratory Measurements

An automated immunoturbidimetric assay (Dako, Glostrup, Denmark) was used to measure serum DBP concentration. The assay was configured for the Roche Modular P System (Roche Diagnostics, Laval, Quebec, Canada). The assay was linear within the calibration range (28 to 544 mg/l), and the measurable range could be expanded with sample dilution. The imprecision of the DBP mass assay was 2.2% at 275 mg/l (Dako Low QC) and 1.4% at 365 mg/l (in-house quality control). Comparison with an established binding capacity assay showed close correlation (*r* = 0.86, *p*<0.0001). Serum total 25OHD and PTH were measured using the Liaison (DiaSorin, Stillwater, Minnesota, US) autoimmunoanalyzer, using chemiluminescent immunoassay technology. Details of the assay were described previously [55]. Estimated glomerular filtration rate was calculated using the Chronic Kidney Disease Epidemiology Collaboration formula [56].

### Genotyping

Genotyping was performed using the Applied Biosystems (ABI) TaqMan protocol. The SNP rs2282679 had a minor allele frequency of 0.288, with no evidence for deviation from Hardy–Weinberg equilibrium (*p* = 0.89). Three other 25OHD-level-associated SNPs previously implicated in GWASs [26], rs12785878 (11q12 near *DHCR7*), rs10741657 (11p15 near *CYP2R1*), and rs6013897 (20q13 near *CYP24A1*), were also genotyped.

### Linear Regression of SNP and DBP Level

All statistical analyses were undertaken using the STATA software package (version 11, StataCorp, College Station, Texas, US). Descriptive statistics of DBP, relevant covariates, and outcomes were assessed. We visually inspected for normality in the distribution of DBP levels in our study population and excluded extreme outliers (>600 mg/l; ∼3.5 standard deviations [SD] from the mean). Further data transformation was not required. A univariable linear regression model was used to assess the association of SNP rs2282679 (coded as 0, 1, and 2, as the count of the C allele) with DBP level (response variable). Results were then adjusted using a multivariate model that included socio-demographic characteristics: age, sex, and ethnicity (European or non-European ancestry).

### Observational Regression Analysis of DBP Level and Clinical Phenotypes

To explore the effect of DBP on phenotypes (common diseases and related traits), we first visually inspected for normality in the distribution of all continuous variables and excluded any extreme outliers (∼3.5 SD from the mean; <1% of all data points). Logarithmic transformation was performed for PTH and fasting insulin. The native distribution was used for 25OHD, calcium, BMI, and BMD. We then generated linear regression models for continuous outcomes (e.g., BMI) and logistic regression models for binary outcomes, adjusted for age and sex. Effect estimates were presented per 1-SD (50 mg/l) increase in DBP. Missing data points were assumed to be missing at random; thus, complete case analyses were performed. We used the adult and youth cohorts to determine the associative effect of DBP on clinical and laboratory-measured traits, i.e., circulating 25OHD, PTH, calcium, fasting glucose and fasting insulin levels, and BMI. As the youth cohort had low risk for the common diseases in question (i.e., myocardial infarction, diabetes, hypertension, and cerebrovascular accidents), we explored the effect of DBP on diseases only in the adult cohort.

### Multiple Variable Regression of SNP and Phenotypes

We then examined the association of SNP rs2282679 with the phenotypes (common diseases and related traits) using linear regression for continuous variables and logistic regression for binary variables, adjusting for age and sex. The null hypothesis was tested using α = 0.05. The Bonferroni method was used to maintain family-wise error rate when performing multiple testing through enforcing a more stringent α for declaring statistical significance for each comparison (0.05/12 = 0.004 for 12 comparisons). Conservatively, independence between outcomes was assumed for this correction.

### Instrumental Variable Analysis of DBP and Clinical Phenotypes

We used the SNP as an IV to estimate the causal effect of DBP on the same outcome measures. For continuous outcomes we used the two-stage least-squares estimator method that regressed each outcome against predicted values of DBP level per genotype using the command “ivreg2” in the STATA software package. The Durbin-Wu-Hausman chi-square test [57], a test for endogeneity in a regression estimated via IVs, was computed using the command “ivendog,” where the null hypothesis states that an ordinary least-squares estimator of the same equation would yield consistent estimates. For dichotomous outcomes (e.g., myocardial infarction), we conducted a two-stage IV analysis. The first stage was a linear regression of the intermediate phenotype (DBP) on the categorical instrument (SNP) to generate DBP fitted values. The second stage was a logistic regression of the clinical outcome on predicted values of the intermediate phenotype, adjusted for age, sex, and the estimated residuals that may be correlated with unmeasured confounders. IV analysis for dichotomous outcomes has been described previously [58].

### Single SNP Look-Ups in GWAS International Consortia

We sought replication of the associations of rs2282679 with these phenotypes in publically available GWAS meta-analysis data from large international consortia that provided single SNP look-up results. Results for this SNP were provided for the following phenotypes: BMI from GIANT [50], BMD from GEFOS [46], type 2 diabetes from DIAGRAM [47], blood pressure measurements from ICBP [48], ischemic stroke from METASTROKE/ISGC [45], and fasting insulin and fasting glucose from MAGIC [49]. Using this SNP (instrument) as the proxy measure in genetic association analysis, these large consortia provided at least 90% power to detect direct associations between rs2282679 (proxy exposure) and continuous outcome variables given a 2% change in the mean of the outcome variable per 1-SD change in DBP with type 1 error rate = 0.004 [59]. Power estimations are shown in Tables S1 and S2.

### Association Analysis of Three Other Vitamin-D-Associated SNPs with Phenotypes

We used univariable linear regression models to determine the association of three other vitamin-D-associated SNPs, rs12785878, rs10741657, and rs6013897, with 25OHD level, and evaluated the association of all four SNPs, including rs2282679, with 25OHD level in a multivariate regression model adjusting for age, sex, ethnicity, and season of blood draw. We then performed single SNP look-ups for these three additional genetic variants in the GWAS meta-analysis data for the same traits and diseases that were tested in CaMos.

## Results

**Figure 1:**
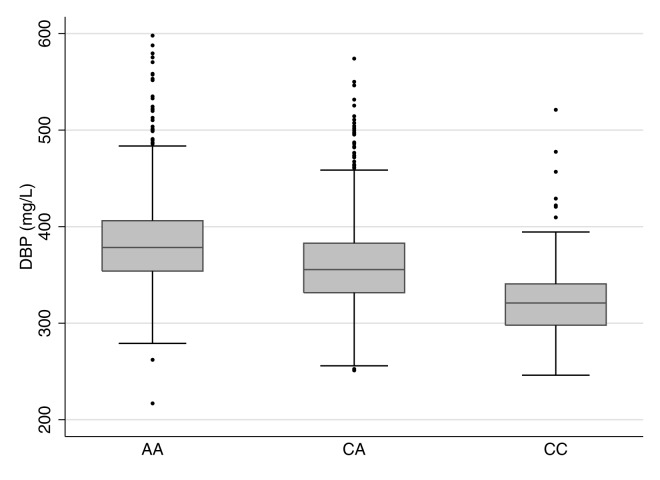
Boxplot of vitamin D binding protein levels by rs2282679 genotype. rs2282679 has three genotypes, i.e., AA, AC, and CC. Homozygous carriers of the major allele (AA) comprised 51.4% of the population, heterozygous carriers (AC) comprised 39.6%, and homozygous carriers of the minor allele (CC) comprised 9.0%. Individuals carrying the effect allele, C, had lower DBP levels than those with the more common allele (AA: 384.6 mg/l [SD 48.3], *n* = 1,159; CA: 360.7 mg/l [SD 45.9], *n* = 893; CC: 322.9 mg/l [SD 39.1], *n* = 202). The effect allele showed an inverse linear relationship with DBP levels.

We tested the relationships between rs2282679, DBP, and 25OHD in 2,254 CaMos participants; 69.5% were women, and 79.5% were of European descent. The mean age was 65.7 (SD 15.4) y, and mean BMI was 27.5 (SD 5.4) kg/m^2^ at the time of clinical assessment. The C allele at rs2282679 had a population frequency of 0.288; genotype frequencies were 0.514 (AA), 0.396 (AC), and 0.090 (CC) (Table 1). We refer to the C allele as the effect allele. Individuals carrying the effect allele had lower DBP levels than those with the more common allele (AA: 384.6 mg/l [SD 48.3], *n* = 1,159; CA: 360.7 mg/l [SD 45.9], *n* = 893; CC: 322.9 mg/l [SD 39.1], *n* = 202). The effect allele showed an inverse linear relationship with DBP level (Figure 1). Univariable linear regression demonstrated a strong relationship between rs2282276 and DBP level (each copy of the effect allele, C, was associated with a decrease of 27.6 [95% CI 24.8, 30.4] mg/l in DBP levels, *p* = 3.3×10^−76^). The rs2282679 SNP explained 14.1% of the variance in DBP level (coefficient of determination, *r* ^2^ = 0.141, *F*-statistic = 368.7). Adjusted for age, sex, and ethnicity, the effect size of this SNP on DBP was similar (−27.4 [95% CI −30.0, −24.7] mg/l); the variance explained was 25%, while the *F*-statistic was 188.7.

**Table 1:** Participant characteristics for the CaMos cohort.

Characteristic	Value
**Participant characteristics (** ***n*** ** = 2,254)**	
Female	1,567 (69.5%)
Age—years	65.72 (SD 15.4)
BMI—kg/m^2^ ◊	27.49 (SD 5.4)
European descent	2,133 (94.6%)
Education beyond high school*	1,183 (52.5%)
No sunlight exposure in last year*	1,151 (51.1%)
Ever smoker*	1,062 (47.1%)
Cold season blood draw¶	1,243 (55.2%)
**rs2282679 polymorphism (** ***n*** ** = 2,254)**	
A/A	1,159 (51.4%)
A/C	893 (39.6%)
C/C	202 (9.0%)
**Laboratory measurements (normal range) (** ***n*** ** = 2,254)**	
BMD at femoral neck—g/cm^2^	0.741 (SD 0.131)
Estimated GFR—ml/min (≥60)	78.9 (SD 19.1)
25OHD—nmol/l (≥75)	68.9 (SD 24.7)
Free 25OHD—pmol/l	14.4 (SD 5.1)
DBP—mg/l	369.6 (SD 50.1)
Calcium—mmol/l (2.13–2.60)	2.38 (SD 0.11)
Albumin—g/l (34–48)	44.2 (SD 2.6)
PTH—ng/l (22.2–108.9)∥	62.4 (SD 30.6)
Fasting glucose—mmol/l (3.3–6.0)	5.51 (SD 1.11)
Fasting insulin—pmol/l	65.4 (SD 44.6)
**Diseases** ° **(** ***n*** ** = 2,122) (adults only)**	
Hypertension	916 (43.2%)
Diabetes	201 (9.5%)
Myocardial infarction	192 (9.0%)
Stroke/TIA	161 (7.6%)
Osteoporosis	475 (22.8%)

Continuous traits are presented as mean (SD), while dichotomous traits are presented as *n* (percent). Unless otherwise stated, characteristics had <2% missing data.

◊ Missing data for 87 (3.9%) participants.

*Education beyond high school, sunlight exposure, and smoking were ascertained in the adult cohort only.

¶ Cold season blood draws are laboratory blood tests performed between November and April inclusively.

∥ Missing data for 171 (7.6%) participants.

° Disease data were obtained only among those genotyped in the adult cohort (*n* = 2,122); GFR, glomerular filtration rate.

While DBP level was associated with age, sex, and ethnicity (Table 2), rs2282679 was not associated with age, sex, ethnicity, or any of the following potential confounders: education beyond high school, sunlight exposure, and smoking status (Table S3). Thus, rs2282679 remained a suitable genetic instrument for DBP level in MR analyses. The association for rs2282679 remained robust (−26.5 [95% CI −29.2, −23.8] mg/l) after additionally adjusting for other potential confounders (i.e., season of blood draw, education beyond high school, sunlight exposure in the last year, and smoking status) (Table S4). This SNP also explained 1.5% of the variance in 25OHD level (*F-*statistic = 34.6).

**Table 2:** Unadjusted and multiple-adjusted linear regression association of rs2282679 with vitamin D binding protein serum concentrations.

Model	Variable	Change in DBP (mg/l) (95% CI)	*p*-Value	*r* ^2^	*F-*Statistic
Univariable model (*n* = 2,248)	rs2282679 (C)	−27.6 (−30.4, −24.8)	3.3×10^−76^	0.14	368.7
Multivariate model (*n* = 2,247)	rs2282679	−27.4 (−30.0, −24.7)	1.3×10^−84^	0.26	197.3
	Female	29.2 (25.4, 32.9)	2.7×10^−50^		
	Age—years	−0.6 (−0.7, −0.5)	3.4×10^−26^		
	Age—decades	−6.2 (−7.3, −5.0)			
	Non-European	−16.9 (−24.9, −9.0)	2.8×10^−5^		

A fully expanded multivariate model for rs2282679 and DBP concentration including additional social factors and laboratory and clinical variables is available in Table S3.

There was no evidence of non-linearity in the relationship between DBP concentration and 25OHD concentration (Figure S1). Observational analysis demonstrated DBP levels to be strongly associated (*p*≤0.004) with changes in 25OHD levels (5.0 [95% CI 3.9, 6.1] nmol/l per 1-SD increase in DBP), calcium levels (0.01 [95% CI 0.01, 0.02] mmol/l per 1-SD increase in DBP), and BMI (−0.5 [95% CI −0.7, −0.3] kg/m^2^ per 1-SD increase in DBP) (Table 3). Weak evidence for association (*p*≤0.1) was observed between DBP and fasting glucose (−0.001 [95% CI −0.002, −7.2×10^−5^] mmol/l per 1-SD increase in DBP), diabetes (odds ratio [OR] 0.79 [95% CI 0.66, 0.95]), hypertension (OR 0.91 [95% CI 0.82, 1.01]), and stroke/transient ischemic attack (TIA) (OR 0.83 [95% CI 0.68, 1.02]). No association was found with the other phenotypes tested, i.e., fasting insulin, PTH, BMD, osteoporosis, and myocardial infarction (*p*>0.1).

**Table 3:** Observational and instrumental variable analyses for the causal association of vitamin D binding protein with 25-hydroxy-vitamin D and common diseases and related traits in the CaMos cohort.

Outcome	Observational Regression Analysis		IV Analysis		Test for Endogeneity *p*-Value*
	Effect Estimate per 50 mg/l of DBP (95% CI)	*p*-Value	Effect Estimate per 50 mg/1 of DBP (95% CI)	*p*-Value	
**Trait (youth and adult cohorts; up to ** ***n*** ** = 2,254)**					
25OHD—nmol/l	4.99 (3.91, 6.07)	3.2×10^−19^	8.17 (5.42, 10.91)	5.5×10^−9^	0.01
Free 25OHD—pmol/l	−0.71 (−0.94, −0.47)	3.1×10^−9^	−0.11 (−0.71, 0.49)	0.49	0.03
Calcium—mmol/l	0.01 (0.01, 0.02)	1.5×10^−5^	0.01 (−0.003, 0.02)	0.12	0.87
PTH∥—ng/l	−0.01 (−0.03, 0.01)	0.21	0.02 (−0.03, 0.07)	0.34	0.11
Fasting glucose—mmol/l	−0.001 (−0.002, −7.2×10^−5^)	0.03	−3.6×10^−4^ (−0.02, 0.02)	0.73	0.59
Fasting insulin∥—pmol/l	−0.02 (−0.05, 0.01)	0.20	0.06 (−0.01, 0.13)	0.11	0.02
BMI—kg/m^2^	−0.53 (−0.76, −0.29)	9.1×10^−6^	0.15 (−0.43, 0.72)	0.61	0.01
BMD at femoral neck—g/cm^2^	0.002 (−0.003, 0.007)	0.46	−0.005 (−0.02, 0.01)	0.43	0.23
**Disease (adult cohort only; up to ** ***n*** ** = 2,122)**					
Stroke/TIA	0.83 (0.68, 1.02)	0.08	0.51 (0.32, 0.81)	0.004	—
Myocardial infarction	0.90 (0.75, 1.08)	0.25	0.80 (0.53, 1.21)	0.29	—
Diabetes	0.79 (0.65, 0.96)	0.02	1.00 (0.66, 1.53)	0.98	—
Hypertension	0.91 (0.82, 1.01)	0.09	0.82 (0.64, 1.05)	0.11	—
Osteoporosis	0.96 (0.85, 1.09)	0.53	1.32 (0.97, 1.79)	0.07	—

Effect estimates are presented as absolute changes for continuous traits or ORs for disease per 1-SD increase in DBP. All observational regression and IV models were adjusted for age and sex only. Analyses of disease conditions were restricted to the adult cohort only. The null hypothesis is tested using α = 0.05. The Bonferroni method is used to maintain family-wise error rate when performing multiple testing through enforcing a more stringent α = 0.05/12 = 0.004 (12 comparisons).

*The Durbin-Wu-Hausman chi-square test (test for endogeneity) was computed for continuous outcomes.

∥ The outcome was transformed using the natural logarithm, and effect estimates are reported accordingly.

¶ Effect estimates with and without excluding participants with diabetes were similar for fasting glucose. Excluding participants with diabetes, effect estimate per 50 mg/l of DBP: −1.3×10^−4^ (95% CI −0.001, 3.9×10^−4^), *p* = 0.63, and 0.001 (95% CI −0.001, 0.002), *p* = 0.47, test for endogenicity, *p* = 0.33; they were also similar for fasting insulin. Excluding participants with diabetes, effect estimate per 50 mg/l of DBP: −2.4×10^−4^ (95% CI −0.001, 3.4×10^−4^), *p* = 0.42, and 0.001 (95% CI −1.9×10^−4^, 0.003), *p* = 0.09, test for endogenicity, *p* = 0.03.

### Mendelian Randomization Analyses in the CaMos Cohort

Using the SNP for MR analyses, DBP levels were shown to have a causal effect on only 25OHD levels (8.2 [95% CI 5.2, 10.9] nmol/l per 1-SD increase in DBP). The other observational associations described above did not persist in the IV analysis, suggesting that associations from the observational regression analyses were non-causal. Assessment for endogeneity indicated that IV testing was particularly meaningful for estimating effects of DBP on 25OHD level (*p* = 0.01), BMI (*p* = 0.01), and insulin level (*p* = 0.02). A suggestive causal association between DBP level and stroke/TIA was found, but this association did not survive multiple-testing correction (OR 0.51 [0.32, 0.81], *p* = 0.004).

In a subgroup analysis, we explored the relationship between DBP and traits using the same IV analysis restricted to CaMos participants with 25OHD levels <50 nmol/l (*n* = 498). In this subgroup, we found that a similar genotype–phenotype relationship was preserved (−27.0 [95% CI −32.4, −21.6] mg/l change in DBP for each copy of the effect allele, C, at rs2282679; *p* = 4.8×10^−21^; *r* ^2^ = 0.16, *F*-statistic = 97.2). Among all the traits tested, higher DBP level was found to be associated only with lower calculated free 25OHD level (−1.1 [95% CI −1.3, −0.9] pmol/l for every 1-SD increase in DBP) in the observational regression analysis; this relationship persisted in the IV analysis (−1.7 [95% CI −2.2, −1.2] pmol/l per 1-SD increase in DBP) and genetic association analysis (0.9 [95% CI 0.6, 1.2] pmol/l per copy of the effect allele).

### Single SNP Look-Up from Large-Scale GWAS International Consortia

In order to provide more precise effect estimates in MR analyses for any particular disease or trait, we replicated our findings of no association between rs2282679 and common diseases and traits using data from the large-scale international GWAS consortia outlined in Table 4. Each of these consortia had adequate power to test our findings at smaller effect sizes.

**Table 4:** rs2282679 polymorphism association with diseases and traits that have been observationally related to vitamin D levels.

CaMos Results				GWAS Meta-Analysis Results			
Phenotype	CaMos Effect Estimate∫	*p*-Value	Sample Size	GWAS Effect Estimate∫	*p*-Value	Sample Size (Cases/Controls)	Consortium
**Disease-related trait**							
25OHD—nmol/l	−4.48 (−6.00, −2.97)	7.3×10^−9^	2,254	—	1.9×10^−109^	33,996	SUNLIGHT
Free 25OHD—pmol/l	−0.06 (−0.27, 0.39)	0.72	2,254	—	—	—	No consortium available
Calcium—mmol/l	−0.005 (−0.012, 0.001)	0.12	2,250	—	—	—	No consortium available
PTH∥—ng/l	−0.01 (−0.04, 0.01)	0.34	2,083	—	—	—	No consortium available
Fasting glucose—mmol/l	0.01 (−0.05, 0.07)	0.73	2,249	0.00 (−0.01, 0.01)	0.997	46,186	MAGIC
Fasting insulin∥—pmol/l	−0.03 (−0.07, 0.01)	0.11	2,178	−0.01 (−0.01, 0.003)	0.22	46,186	MAGIC
BMI—kg/m^2^	−0.08 (−0.39, 0.23)	0.63	2,167	0.00 (−0.01, 0.01)	0.80	127,587	GIANT
BMD at femoral neck—g/cm^2^	0.003 (−0.004, 0.01)	0.43	2,213	0.01 (−0.01, 0.03)	0.36	32,961	GEFOS
Mean arterial pressure—mm Hg	—	—	—	−0.06 (−0.19, 0.07)	0.36	28,775	ICBP
**Disease**							
Stroke/TIA	1.45 (1.14, 1.85)	0.003	2,122	1.00 (0.97, 1.04)|	0.92	12,389/62,004	METASTROKE/ISGC
Coronary artery disease	1.13 (0.90, 1.42)	0.30	2,122	1.02 (0.99, 1.05)	0.31	22,233/64,762	CARIDoGRAM
Diabetes	0.99 (0.79, 1.24)	0.93	2,122	1.01 (0.97, 1.05)	0.76	9,580/53,810	DIAGRAM
Hypertension	1.12 (0.98, 1.28)	0.11	2,116	—	—	—	ICBP

Regression models were adjusted for age and sex. Analyses of disease-related traits were performed on both adult and youth CaMos cohorts; analyses of diseases were restricted to the CaMos adult cohort only. M-dashes indicate that summary data were not publically available.

∫ Measurement change for disease-related traits and OR for diseases. Change in measurement of disease-related trait is per each additional copy of the risk allele. All continuous variables were inspected for normality, and outliers (<1% of data points) were excluded. For diseases, ORs are reported per copy of risk allele. The null hypothesis is tested using α = 0.05. The Bonferroni method is used to maintain family-wise error rate when performing multiple testing through enforcing a more stringent α = 0.05/12 = 0.0042 (12 comparisons). Independence between outcomes was assumed in this correction.

∥ The natural logarithm was used.

¶ Effect estimates excluding participants with diabetes were similar for fasting glucose, −0.013 (95% CI −0.050, 0.023), *p* = 0.47, and for fasting insulin, −0.034 (95% CI −0.075, 0.007), *p* = 0.10.

* ICBP had available GWAS data only on blood pressure measurements, not on hypertension; no association was found for systolic blood pressure, diastolic blood pressure, mean arterial pressure, and pulse pressure.

| This effect estimate provided by METASTROKE is for overall ischemic stroke, which does not include TIA.

SNP rs2282679 was found to be strongly associated with 25OHD level in SUNLIGHT (*n* = 33,996, *p* = 1.9×10^−109^). However, there was no association between this SNP and other common diseases and traits tested, and, importantly, the confidence intervals for each association were tightly centered at the null. Thus, our findings suggest a lack of causal effect of DBP level on these traits and diseases.

### Association of Three Other Vitamin-D-Associated SNPs with Phenotypes

In CaMos, although rs2282679 demonstrated the strongest association with 25OHD across all SNPs tested, two other SNPs involved in vitamin D metabolism (rs12785878 [11q12 near *DHCR7*] and rs10741657, [11p15 near *CYP2R1*]) were also found to be associated with 25OHD level, with or without adjustment for age, sex, ethnicity, and season of blood draw. A third SNP, rs6013897 (20q13 near *CYP24A1*), also previously implicated in vitamin D metabolism, was not significantly associated with 25OHD level (Table S6). Single SNP look-ups in large GWAS meta-analysis results (Table S7) revealed that all three SNPs were strongly associated with 25OHD level in SUNLIGHT (rs12785878: *p* = 2.1×10^−27^; rs10741657: *p* = 3.3×10^−20^; rs6013897, *p* = 6.0×10^−10^). rs6013897 was also found to be associated with BMD measurements at a threshold below genome-wide significance in GEFOS (*p* = 3.04×10^−5^). We observed no other associations between these SNPs and other calcemic or cardiometabolic traits.

## Discussion

Our study documented a strong association between SNP rs2282679 and both serum DBP and 25OHD levels. As this SNP explained a large proportion of the variance in DBP levels, it enabled us to use this polymorphism in causal IV analyses to demonstrate that DBP levels are unlikely to have a causal role in all other tested traits and diseases. These observations, from large sample sizes, suggest that if 25OHD level does influence risk of these diseases, DBP level has little role in mediating this effect.

Each additional copy of the effect allele at rs2282679 lowered DBP concentration by 27.6 mg/l (95% CI 24.8, 30.4; *p* = 3.25×10^−76^) and explained 14.1% of its variance. Further, this effect allele was strongly correlated with reduced circulating 25OHD concentration (−4.3 nmol/l [95% CI −5.9, −2.7]; *p* = 8.0×10^−8^) in our study population and explained 1.5% of the variance in 25OHD level; only an additional 1.1% was explained by the other three vitamin-D-related SNPs, and another 3.5% by socio-demographic and environmental factors (Table S6). SNP rs2282679 may thus define genetic susceptibility to vitamin D deficiency in certain individuals and may also partly explain the differential increments in circulating 25OHD in response to vitamin D supplementation and ultraviolet light exposure. This effect allele was common in the population (minor allele frequency = 0.29) and explained a large proportion of the variance in DBP level, which highlights a direct mechanism of action between the gene and circulating levels of the protein and provides an ideal IV for a MR study. Results from our study indicate that DBP was causally responsible for circulating 25OHD level (*p* = 3.16×10^−19^).

Given that serum 25OHD concentration has an inverse relationship with PTH concentration, PTH level can be used as a functional readout for 25OHD level. Increased 25OHD levels are associated with increased calcium absorption, which may increase serum calcium and decrease production and secretion of PTH. Increased 25OHD levels may also result in augmented renal and/or parathyroid conversion of 25OHD to 1,25-dihydroxy-vitamin D (1,25OH_2_D). The consequent increase in either systemic or local production of 1,25OH_2_D can decrease PTH gene expression, and reduce PTH production [60],[61]. However, while DBP level strongly correlates with 25OHD level, our results indicate that DBP concentration does not influence PTH concentration. We recently reported that, in this largely vitamin-D-sufficient cohort, while total and free 25OHD levels correlated negatively with PTH, this relationship is mainly independent of DBP concentration [61]. In mice with targeted deletion of the *Gc* gene that completely lack DBP, PTH levels were not different between controls and animals without vitamin D toxicity or prolonged vitamin D deficiency (25OHD ≤6 ng/ml [15 nmol/l]) [23]. Our current MR study supports these findings. A recent study by Powe and colleagues [62] reported that only slightly higher PTH levels were found among black Americans with lower total 25OHD concentrations and DBP concentrations than among white Americans with lower total 25OHD concentrations and DBP concentrations, consistent with our findings. Powe and colleagues also identified that the large differences in 25OHD levels observed between black and white Americans are likely due to differences in DBP levels, which, once accounted for, lead to similar levels of bioavailable 25OHD in the two ethnic groups [62]. While these findings may have considerable impact on definitions of vitamin D deficiency, findings from our study do not provide evidence that differences in DBP levels impact risk of the common diseases tested.

MR studies have several advantages over observational studies. As genetic variants are stable throughout life, reverse causality and other forms of biases commonly associated with observational studies are removed; this strengthens causal associations between exposures and outcomes [63]. Indeed, given that the distribution of the genetic variation in the population is largely a random process (i.e., random assortment at meiosis), conclusions from MR studies can be likened to those from randomized controlled trials. In our study, the point effect estimate of DBP level on 25OHD level (8.39 nmol/l [95% CI 5.55, 11.22]) from our IV analysis was larger than the observational effect estimate (5.03 nmol/l [95% CI 3.92, 6.14]; test for endogeneity *p* = 0.01), suggesting that circulating 25OHD concentration may depend even more strongly on DBP level than as is seen in observational studies. Conversely, the similar effect estimates from IV and observational analyses for fasting glucose, BMD, calcium, and PTH suggest that these observational associations are unlikely to be biased by confounding or reverse causality. Indeed, this MR analysis has further refined our understanding of the clinical significance of DBP as a biomarker, and demonstrates that this serum protein is not a critical player in a causal pathway potentially linking vitamin D to the common diseases assessed.

While MR is able to strengthen causal inference, it faces potential limitations [49],[64]. Confounding can occur if polymorphisms in linkage disequilibrium with rs2282679 also have phenotypic effects related to the common diseases tested. We were also not able to account for possible pleiotropic effects of *GC* on physiologic pathways that could have masked tested associations. Null results could also be explained by canalization, i.e., phenotypic effects of genetic variations may be buffered during development [65]. Population stratification can also confound genotype–disease relationships if the association is influenced by factors related to population structure. Notably, adjusting for ethnicity in our regression models did not change the strength of associations. However, given that the overwhelming majority of all populations tested were of European descent, our findings are only applicable to those of European ancestry. Further, all tested populations in international consortia were primarily of European descent, making confounding by population stratification unlikely. We also acknowledge that our results are generalizable only to largely healthy, community-dwelling adults and not to infants, children, pregnanct women, or periods of human growth and development.

Another potential limitation of genetic association studies is unreliable genotype–intermediate phenotype associations [66]. This is unlikely in this case, as not only does rs2282679 explain 14.1% of the variance in DBP level, but it is also strongly associated with DBP level (CaMos, *p* = 3.3×10^−76^, *n* = 2,254; TwinsUK, *p* = 4.0×10^−42^, *n* = 1,674 [26]). While the large variance explained and power anticipated from this single SNP made it an ideal candidate for a genetic instrument for DBP, we acknowledge that this instrument conveys only one aspect of the overall vitamin D status in common diseases, and therefore can assess the causal effect of 25OHD level on common diseases only as mediated by DBP level. Three other SNPs previously implicated in the synthesis or metabolism of vitamin D (rs12785878, rs10741657, and rs6013897) were found to be strongly associated with 25OHD level in SUNLIGHT (rs12785878: *p* = 2.1×10^−27^; rs10741657: *p* = 3.3×10^−20^; rs6013897, *p* = 6.0×10^−10^), but were not associated with any other calcemic or cardiometabolic traits, except for femoral neck BMD. A large MR study on 25OHD level and blood pressure (*n* = 146,581) that used SNPs involved only in the synthesis of vitamin D (rs12785878 and rs12794714) as genetic instruments reported a small but significant association with hypertension (OR 0.92 [95% CI 0.87, 0.97]; *p* = 0.001) and borderline associations with diastolic (−0.29 mm Hg [95% CI −0.52, −0.07] per 10% increase in 25OHD level; *p* = 0.01) and systolic blood pressure (−0.37 mm Hg [95% CI −0.73, 0.003]; *p* = 0.052) [67]. However, the clinical implications of these possible effects have yet to be elucidated.

A study limitation is that biochemical information was cross-sectional, which prevents us from examining the impact of biological variability in DBP level over time on common diseases, and that our study has modeled a linear relationship between DBP and common diseases. Although we were able to replicate our MR findings using large GWAS data for the majority of diseases and traits, we were not able to do so for PTH; thus, smaller effects of DBP on PTH level may remain undetected by our study. Further, while stroke and TIA were determined in CaMos by self-report through a single interview question, METASTROKE had GWAS data only on ischemic stroke and its subtypes, not TIA. Hypertension was ascertained in CaMos by self-report and not blood pressure measurements, whereas ICBP provided publically available GWAS data on blood pressure measurements and not hypertension. Observational regression analyses showed that DBP level was associated with BMI (*p* = 9.1×10^−6^); however, this association was absent in IV analysis (*p* = 0.46), which is consistent with previous large-sale MR studies of BMI and 25OHD level [68] and suggests the presence of an endogenous regressor (*p* = 0.01). Such observational associations require instrumenting as they are potentially spurious because of unmeasured confounders or affected by reverse causality. Likewise, we provide data demonstrating a lack of causal relationship between DBP level and all tested common diseases and traits, except 25OHD level, from large and well-powered international consortia.

Some evidence suggests that 25OHD level may exert different threshold effects on PTH level and on skeletal outcomes. High serum concentrations of 25OHD (>100 nmol/l) have markedly diminished effects on PTH level [55]. A meta-analysis of effects of vitamin D supplementation by Reid and colleagues showed improvement in BMD at the femoral neck mainly in individuals with baseline 25OHD <50 nmol/l and not >50 nmol/l [69]. It is therefore possible that 25OHD may have an appreciable impact on non-skeletal outcomes only at levels <50 nmol/l. However, we note that among CaMos participants, DBP level has no obvious threshold effect (or any other non-linear relationship) on 25OHD or log-PTH levels (Figures S1 and S2, respectively).

In our subgroup analysis of participants with 25OHD levels <50 nmol/l, among all the traits tested, DBP was causally associated only with calculated free 25OHD level. Importantly, calculated free 25OHD level is assessed using DBP level, and thus this association is to be interpreted with caution. A future study using laboratory-measured bioavailable 25OHD level can be performed to test whether these associations can be replicated in larger cohorts of vitamin-D-deficient participants.

In sum, this MR study, with replication of null findings in large-scale GWAS meta-analyses, demonstrates that while a strong causal relationship exists between serum DBP and 25OHD level, DBP level is not causally associated with any of the calcemic and cardiometabolic common diseases tested. This suggests that if 25OHD does have causal influence on common diseases in the general population, it likely acts in a manner independent of DBP. Our results are generalizable to other vitamin-D-replete populations of community-dwelling healthy adults where the majority have 25OHD levels >50 nmol/l [70],[71]. Three other genetic variants that strongly influence 25OHD level, and influence 25OHD metabolism in ways independent of DBP, also showed no effect upon cardiometabolic disease. It remains to be determined whether 25OHD has a causal effect on these outcomes independent of DBP.

## Supporting Information

Figure S1 Scatter plot of 25-hydroxy-vitamin D levels and vitamin D binding protein levels.
(DOCX)

Figure S2 Scatter plot of parathyroid hormone levels and vitamin D binding protein levels.
(DOCX)

Table S1 Power calculation for rs2282679 association with dichotomous outcomes in CaMos and in GWAS meta-analyses.
(DOCX)

Table S2 Power calculation for Mendelian randomization analyses for DBP and continuous traits using rs2282679 as the genetic instrument.
(DOCX)

Table S3 Association between the genetic instrument, rs2282679, and three other vitamin-D-associated SNPs, rs12785878, rs10741657, and rs6013897, with potential confounders in the CaMos cohort.
(DOCX)

Table S4 Fully expanded, multiply adjusted linear regression of rs2282679 polymorphism and vitamin D binding protein serum concentration.
(DOCX)

Table S5 Observational regression and instrumental variable analyses for the causal association of vitamin D binding protein with disease-related traits among CaMos participants with 25-hydroxy-vitamin D levels less than 50 nmol/l.
(DOCX)

Table S6 Unadjusted and multiple-adjusted linear regression association of rs2282679, rs12785878, rs10741657, and rs6013897 with 25-hydroxy-vitamin D serum concentration.
(DOCX)

Table S7 Association of vitamin-D-associated SNPs rs12785878, rs10741657, and rs6013897 with diseases and related traits, from GWAS meta-analyses.
(DOCX)

## Abbreviations

1,25OH_2_D: 1,25-dihydroxy-vitamin D
25OHD: 25-hydroxy-vitamin D
BMD: bone mineral density
BMI: body mass index
CaMos: Canadian Multicentre Osteoporosis Study
CARDIoGRAM: Coronary Artery Disease Genome Wide Replication and Meta-analysis
DBP: vitamin D binding protein
DIAGRAM: Diabetes Genetics Replication and Meta-analysis
GEFOS: Genetic Factors for Osteoporosis Consortium
GIANT: Genetic Investigation of Anthropometric Traits
GWAS: genome-wide association study
ICBP: International Consortium for Blood Pressure
ISGC: International Stroke Genetics Consortium
IV: instrumental variable
MAGIC: Meta-analyses of Glucose and Insulin-Related Traits Consortium
MR: Mendelian randomization
OR: odds ratio
PTH: parathyroid hormone
SD: standard deviation
SNP: single nucleotide polymorphism
SUNLIGHT: The Study of Underlying Genetic Determinants of Vitamin D and Highly Related Traits
TIA: transient ischemic attack
