# Supplementary material for: The Causal Effect of Vitamin D Binding Protein (DBP) Levels on Calcemic and Cardiometabolic Diseases: A Mendelian Randomization Study
Source: PLoS Med. 2014 Oct 28;11(10):e1001751. doi: 10.1371/journal.pmed.1001751 (PMC4211663; doi:10.1371/journal.pmed.1001751)
Supplement: Figure S1 — Scatter plot of 25-hydroxy-vitamin D levels and vitamin D binding protein levels. (DOCX) [file pmed.1001751.s001.docx]

**Figure S1: Scatter plot of 25-hydroxy-vitamin D (25OHD) levels and vitamin D binding protein (DBP) levels**
