# Supplementary material for: The Causal Effect of Vitamin D Binding Protein (DBP) Levels on Calcemic and Cardiometabolic Diseases: A Mendelian Randomization Study
Source: PLoS Med. 2014 Oct 28;11(10):e1001751. doi: 10.1371/journal.pmed.1001751 (PMC4211663; doi:10.1371/journal.pmed.1001751)
Supplement: Figure S2 — Scatter plot of parathyroid hormone levels and vitamin D binding protein levels. (DOCX) [file pmed.1001751.s002.docx]

**Figure S2: Scatter plot of parathyroid hormone (PTH) levels and vitamin D binding protein (DBP) levels.**
