# Supplementary material for: The Causal Effect of Vitamin D Binding Protein (DBP) Levels on Calcemic and Cardiometabolic Diseases: A Mendelian Randomization Study
Source: PLoS Med. 2014 Oct 28;11(10):e1001751. doi: 10.1371/journal.pmed.1001751 (PMC4211663; doi:10.1371/journal.pmed.1001751)
Supplement: Table S1 — Power calculation for rs2282679 association with dichotomous outcomes in CaMos and in GWAS meta-analyses. (DOCX) [file pmed.1001751.s003.docx]

**Table S1: Power calculation for rs2282679 association with dichotomous outcomes in CaMos and in GWAS meta-analyses.**

|  | **CaMos study** | | **GWAS meta-analysis data** | | |
| --- | --- | --- | --- | --- | --- |
| **Disease** | **Estimated power** | **Sample size** | **Estimated power** | **Sample size (Cases/controls)** | **Consortium** |
| Stroke and TIA | 80% | 2,122 | 93% | 12,389/62,004 | METASTROKE/ISGC |
| Coronary Artery Disease | 89% | 2,122 | 100% | 22,233/64,762 | CARIDoGRAM |
| Diabetes | 90% | 2,122 | 85% | 9,580/53,810 | DIAGRAM |
| Hypertension | 100% | 2,116 | - | - | ICBP |

For GWAS meta-analysis data, power calculations assumed an odds ratio of 1.04 per effect allele for dichotomous outcomes and disease prevalence of 10%. For CaMos data, power calculations assumed an odds ratio of 1.5 per effect allele. Sample sizes: Maximum sample sizes indicated either in published paper or publically available summary data. ICBP made available GWAS meta-analysis results for blood pressure as a continuous trait, therefore power calculation for this trait is displayed in Table S2.
