# Supplementary material for: The Causal Effect of Vitamin D Binding Protein (DBP) Levels on Calcemic and Cardiometabolic Diseases: A Mendelian Randomization Study
Source: PLoS Med. 2014 Oct 28;11(10):e1001751. doi: 10.1371/journal.pmed.1001751 (PMC4211663; doi:10.1371/journal.pmed.1001751)
Supplement: Table S2 — Power calculation for Mendelian randomization analyses for DBP and continuous traits using rs2282679 as the genetic instrument. (DOCX) [file pmed.1001751.s004.docx]

**Table S2: Power calculation for Mendelian Randomization analyses for DBP on continuous traits using rs2282679 as the genetic instrument.**

| **Phenotypes** | **Sample size** | **Estimated power** | **Sample size (cases/controls)** | **Estimated power** | **Consortium** |
| --- | --- | --- | --- | --- | --- |
| **Disease-related Traits** |  |  |  |  |  |
| 25OHD - nmol/L | 2,254 | 100% | 33,996 | 91% | SUNLIGHT |
| Calcium – mmol/L | 2,250 | 100% | - |  | No consortium available |
| PTH^║^ - ng/L | 2,083 | 100% | - |  | No consortium available |
| Fasting glucose – mmol/L | 2,249 | 100% | 46,186 | 100% | MAGIC |
| Fasting insulin^║^ – pmol/L | 2,178 | 87% | 46,186 | 100% | MAGIC |
| BMI – kg/m^2^ | 2,167 | 100% | 127,587 | 100% | GIANT |
| BMD at femoral neck – g/cm^2^ | 2,213 | 100% | 32,961 | 100% | GEFOS |
| Mean arterial pressure – mmHg | - |  | 28,775 | 100% | ICBP^┌^ |

For GWAS meta-analysis data, power calculation for Mendelian Randomization analysis assumed a change of 2% of the mean of the continuous trait per 1 SD change in DBP. For CaMos data, power calculation for Mendelian Randomization analysis assumed a change of 15% of the mean of the continuous trait per 1 SD change in DBP; Sample sizes: Maximum sample sizes indicated either in published paper or publically available summary data.
