# Supplementary material for: The Causal Effect of Vitamin D Binding Protein (DBP) Levels on Calcemic and Cardiometabolic Diseases: A Mendelian Randomization Study
Source: PLoS Med. 2014 Oct 28;11(10):e1001751. doi: 10.1371/journal.pmed.1001751 (PMC4211663; doi:10.1371/journal.pmed.1001751)
Supplement: Table S3 — Association between the genetic instrument, rs2282679, and three other vitamin-D-associated SNPs, rs12785878, rs10741657, and rs6013897, with potential confounders in the CaMos cohort. (DOCX) [file pmed.1001751.s005.docx]

**Table S3: Association between the genetic instrument, rs2282679, and three other vitamin D-associated SNPs, rs12785878, rs10741657 and rs6013897, with potential confounders in the CaMos cohort.**

| **SNP** | **Potential confounder** | **Effect estimate per copy of effect allele** | **95% CI** | **P-value** |
| --- | --- | --- | --- | --- |
| **rs2282679** | Age | Difference: 0.36 years | (-0.61, 1.34) | 0.466 |
|  | Sex  (Female vs. male) | Odds ratio (OR): 0.98 | (0.85, 1.12) | 0.742 |
|  | Race  (non-European vs. European) | OR 0.91 | (0.68, 1.21) | 0.507 |
|  | Education  (> high school vs. ≤ high school) | OR 1.00 | (0.88 1.14) | 0.998 |
|  | Sunlight exposure  (≥ some vs. never) | OR 0.98 | (0.86, 1.11) | 0.715 |
|  | Smoker (ever vs. never) | OR 0.93 | (0.82, 1.06) | 0.285 |
| **rs12785878** | Age | Difference: -0.13 years | (-1.12, 0.87) | 0.803 |
|  | Sex  (Female vs. male) | OR 0.91 | (0.79, 1.05) | 0.182 |
|  | Race  (non-European vs. European) | OR 4.07 | (3.10, 5.35) | 0.000 |
|  | Education  (> high school vs. ≤ high school) | OR 1.05 | (0.91, 1.19) | 0.547 |
|  | Sunlight exposure  (≥ some vs. never) | OR 1.05 | (0.92, 1.20) | 0.495 |
|  | Smoker (ever vs. never) | OR 0.98 | (0.86, 1.12) | 0.767 |
| **rs10741657** | Age | Difference: 0.41years | (-0.53, 1.35) | 0.394 |
|  | Sex  (Female vs. male) | OR 0.92 | (0.81, 1.05) | 0.206 |
|  | Race  (non-European vs. European) | OR 0.76 | (0.57, 1.00) | 0.053 |
|  | Education  (> high school vs. ≤ high school) | OR 1.10 | (0.97, 1.24) | 0.158 |
|  | Sunlight exposure  (≥ some vs. never) | OR 1.01 | (0.89, 1.14) | 0.924 |
|  | Smoker (ever vs. never) | OR 0.97 | (0.85, 1.10) | 0.620 |
| **rs6013897** | Age | Difference: 1.62 years | (0.46, 2.78) | 0.006 |
|  | Sex  (Female vs. male) | OR 0.99 | (0.84, 1.16) | 0.868 |
|  | Race  (non-European vs. European) | OR 0.95 | (0.67, 1.33) | 0.748 |
|  | Education  (> high school vs. ≤ high school) | OR 1.15 | (0.98, 1.34) | 0.080 |
|  | Sunlight exposure  (≥ some vs. never) | OR 0.94 | (0.81, 1.10) | 0.454 |
|  | Smoker (ever vs. never) | OR 0.96 | (0.83, 1.13) | 0.649 |

rs12785878 was associated with race and rs6013897 was associated with age. The multivariate genetic model for 25OHD levels (Table S6) adjusted for age, sex, race and season of blood draw. rs2282679: Non-effect allele, A, Effect allele, C; rs12785878: G, T; rs10741657: G, A; rs6013897: T, A.
