# Supplementary material for: The Causal Effect of Vitamin D Binding Protein (DBP) Levels on Calcemic and Cardiometabolic Diseases: A Mendelian Randomization Study
Source: PLoS Med. 2014 Oct 28;11(10):e1001751. doi: 10.1371/journal.pmed.1001751 (PMC4211663; doi:10.1371/journal.pmed.1001751)
Supplement: Table S4 — Fully expanded, multiply adjusted linear regression of rs2282679 polymorphism and vitamin D binding protein serum concentration. (DOCX) [file pmed.1001751.s006.docx]

**Table S4: Fully expanded multiply-adjusted linear regression for rs2282679 polymorphism on vitamin D binding protein (DBP) serum concentration.**

| Variables | Change in DBP (mg/L) | P-value | r^2^ | F | N |
| --- | --- | --- | --- | --- | --- |
| rs2282679 | -26.5 (-29.2, -23.8) | 1.2 x 10^-75^ | 0.26 | 49.7 | 1,941 |
| Female | 26.6 (22.5, 30.8) | 1.4 x 10^-35^ |  |  |  |
| Age – year | -0.7 (-0.9, -0.4) | 2.0 x 10^-09^ |  |  |  |
| Age – decade | -6.6 (-8.7, -4.4) |  |  |  |  |
| Non-European | -15.9 (-23.6, -8.2) | 4.9 x 10^-05^ |  |  |  |
| Cold season blood draw | -2.3 (-5.9, 1.2) | 0.20 |  |  |  |
| > High school education | -2.9 (-6.5, 0.7) | 0.12 |  |  |  |
| Ever smoking | 3.7 (0.2, 7.2) | 0.04 |  |  |  |
| ≥ Some sunlight exposure | -0.1 (-3.7, 3.5) | 0.94 |  |  |  |
| BMI - kg/m^2^ | -0.6 (-0.9, -0.2) | 0.002 |  |  |  |
| BMI - per 5 kg/m^2^ | -2.7 (-4.5, -1.1) |  |  |  |  |
| 25OHD - nmol/L | 0.2 (0.1, 0.3) | 5.7 x 10^-07^ |  |  |  |
| PTH – pg/ml (log) | 0.2 (-4.2, 4.6) | 0.92 |  |  |  |
| Albumin – ng/L | 1.2 (0.4, 2.0) | 0.005 |  |  |  |
| Calcium – mmol/L | 16.4 (-2.7, 35.4) | 0.09 |  |  |  |
| Estimated GFR – ml/min | -0.08 (-0.21, 0.05) | 0.21 |  |  |  |
| BMD at femoral neck – g/cm^2^ | 8.5 (-7.9, 24.9) | 0.31 |  |  |  |

The fully expanded multivariate model adjusts for socio-demographic, laboratory and clinical variables. r^2^: Coefficient of determination; F: F-statistic
