# Supplementary material for: The Causal Effect of Vitamin D Binding Protein (DBP) Levels on Calcemic and Cardiometabolic Diseases: A Mendelian Randomization Study
Source: PLoS Med. 2014 Oct 28;11(10):e1001751. doi: 10.1371/journal.pmed.1001751 (PMC4211663; doi:10.1371/journal.pmed.1001751)
Supplement: Table S5 — Observational regression and instrumental variable analyses for the causal association of vitamin D binding protein with disease-related traits among CaMos participants with 25-hydroxy-vitamin D levels less than 50 nmol/l. (DOCX) [file pmed.1001751.s007.docx]

**Table S5: Observational regression and instrumental variable analyses for the causal association of vitamin D binding protein with disease-related traits among CaMos participants with 25-hydroxy-vitamin D levels less than 50 nmol/L.**

|  | **Observational regression analysis** | | | | **Instrumental variable analysis** | | | | | **Genetic association analysis** | | | |
| --- | --- | --- | --- | --- | --- | --- | --- | --- | --- | --- | --- | --- | --- |
| **Traits** | **Effect estimate** | **LL** | **UL** | **P-value** | **Effect estimate** | **LL** | **UL** | **P-value** | **Endogeneity**  **P-value** | **Effect estimate** | **LL** | **LL** | **P-value** |
| **FN BMD– g/cm^2^** | 0.001 | -0.011 | 0.012 | 0.895 | -0.007 | -0.034 | 0.021 | 0.627 | 0.552 | 0.004 | -0.011 | 0.018 | 0.628 |
| **BMI – kg/m^2^** | -0.408 | -0.940 | 0.124 | 0.132 | 0.278 | -0.996 | 1.551 | 0.669 | 0.245 | -0.146 | -0.819 | 0.526 | 0.669 |
| **Insulin – pmol/L** | -0.050 | -0.115 | 0.015 | 0.132 | 0.127 | -0.037 | 0.291 | 0.129 | 0.017 | -0.065 | -0.147 | 0.017 | 0.121 |
| **Glucose – mmol/L** | -0.094 | -0.191 | 0.002 | 0.056 | -0.113 | -0.344 | 0.119 | 0.339 | 0.862 | 0.059 | -0.063 | 0.182 | 0.343 |
| **Calcium– mmol/L** | 0.019 | 0.008 | 0.029 | 0.001 | 0.005 | -0.022 | 0.031 | 0.727 | 0.255 | -0.002 | -0.016 | 0.012 | 0.729 |
| **Free 25OHD -pmol/L** | -1.097 | -1.299 | -0.896 | 3.8 x10^-24^ | -1.733 | -2.237 | -1.229 | 1.6 x10^-11^ | 0.005 | 0.910 | 0.636 | 1.183 | 1.5x10^-10^ |
| **25OHD - nmol/L** | -0.229 | -1.161 | 0.702 | 0.629 | -2.786 | -5.082 | -0.489 | 0.017 | 0.014 | 1.467 | 0.291 | 2.643 | 0.015 |
| **PTH – ng/L** | 0.016 | -0.027 | 0.059 | 0.459 | 0.024 | -0.081 | 0.128 | 0.658 | 0.879 | -0.012 | -0.067 | 0.042 | 0.660 |

As this subsidiary analysis was restricted only to individuals with 25OHD levels<50 nmol/L, DBP effect estimates on 25OHD levels may be attenuated and should be interpreted with caution. Effect estimate: For observational regression and instrumental variable analyses, change in trait levels for each 1 standard deviation increase in vitamin D binding protein levels; for genetic association analysis, change in trait levels for each additional copy of the effect allele; LL: Lower limit of 95% confidence interval; UL: Upper limit of 95% confidence interval; BMD: Bone Mineral Density; BMI: Body-Mass-Index, 25OHD: 25-hydrox-vitamin D levels; Free 25OHD: calculated free 25OHD levels; PTH: parathyroid hormone levels; PTH and insulin were logarithmically transformed.
