# Supplementary material for: The Causal Effect of Vitamin D Binding Protein (DBP) Levels on Calcemic and Cardiometabolic Diseases: A Mendelian Randomization Study
Source: PLoS Med. 2014 Oct 28;11(10):e1001751. doi: 10.1371/journal.pmed.1001751 (PMC4211663; doi:10.1371/journal.pmed.1001751)
Supplement: Table S6 — Unadjusted and multiple-adjusted linear regression association of rs2282679, rs12785878, rs10741657, and rs6013897 with 25-hydroxy-vitamin D serum concentration. (DOCX) [file pmed.1001751.s008.docx]

**Table S6: Unadjusted and multiple-adjusted linear regression association of rs2282679, rs12785878, rs10741657 and rs6013897 with 25-hydroxy-vitamin D (25OHD) serum concentration.**

|  | **Variables** | **Nearest gene** | **Change in 25OHD, 95% CI (mmol/L)** | **P-value** | **r^2^** | **F-statistic** |
| --- | --- | --- | --- | --- | --- | --- |
| Univariate models |  |  |  |  |  |  |
| (N=2,254) | rs2282679 | *GC* | -4.7 (-6.2, -3.1) | 4.6 x 10^-09^ | 0.015 | 34.6 |
| (N=2,248) | rs6013897 | 20q13 near *CYP24A1* | -1.6 (-3.5, 0.2) | 0.083 | 0.001 | 0.1 |
| (N=2,242) | rs10741657 | 11p15 near *CYP2R1* | 3.1 (1.6, 4.6) | 4.0 x 10^-05^ | 0.007 | 16.9 |
| (N=2,241) | rs12785878 | 11q12 near *DHCR7* | -2.6 (-4.2, -1.0) | 0.001 | 0.004 | 10.6 |
| (N=2,254) | Age – year |  | -0.03 (-0.10, 0.03) | 0.324 | 4 x 10^-04^ | 1.0 |
| (N=2,254) | Female |  | 0.8 (-1.4, 3.1) | 0.455 | 2 x 10^-04^ | 0.6 |
| (N=2,254) | Non-European |  | -6.8 (-11.3, -2.3) | 0.003 | 0.004 | 8.8 |
| (N=2,254) | Season of blood draw |  | 7.1 (5.0, 9.1) | 1.1x10^-11^ | 0.020 | 46.7 |
| (N=2,122) | ≥high school education |  | 3.5 (1.3, 5.6) | 0.001 | 0.004 | 10.3 |
| (N=2,122) | ≥Some sunlight exposure |  | 2.0 (-0.1, 4.1) | 0.066 | 0.001 | 3.4 |
| (N=2,122) | Ever smoking |  | -4.3 (-6.4, -2.2) | 4.9 x 10^-05^ | 0.007 | 16.5 |
| Multivariate genetic model  (N=2,227) | rs2282679 | *GC* | -4.6 (-6.1, -3.0) | 9.8 x 10^-09^ | 0.026 | 16.1 |
|  | rs6013897 | 20q13 near *CYP24A1* | -1.4 (-3.2, 0.5) | 0.143 |  |  |
|  | rs10741657 | 11p15 near *CYP2R1* | 3.1 (1.7, 4.6) | 3.4 x 10^-05^ |  |  |
|  | rs12785878 | 11q12 near *DHCR7* | -2.7 (-4.2, -1.1) | 8.9 x 10^-04^ |  |  |
| Full multivariate model  (n=2,095) | rs2282679 | *GC* | -4.3 (-5.9, -2.7) | 8.0 x 10^-08^ | 0.061 | 13.3 |
|  | rs6013897 | 20q13 near *CYP24A1* | -1.0 (-2.9, 0.9) | 0.288 |  |  |
|  | rs10741657 | 11p15 near *CYP2R1* | 2.9 (1.4, 4.4) | 1.3 x 10^-04^ |  |  |
|  | rs12785878 | 11q12 near *DHCR7* | -2.4 (-4.1, -0.8) | 0.004 |  |  |
|  | Female |  | -0.9 (-3.2, 1.4) | 0.435 |  |  |
|  | Age – year |  | 0.0 (-0.1, -0.1) | 0.515 |  |  |
|  | Non-European |  | -6.3 (-10.9, -1.7) | 0.007 |  |  |
|  | Season of blood draw |  | 7.2 (5.1, 9.3) | 1.2 x 10^-11^ |  |  |
|  | ≥ high school education |  | 3.2 (1.1, 5.3) | 0.003 |  |  |
|  | ≥ sunlight exposure |  | 1.8 (-0.3, 4.0) | 0.090 |  |  |
|  | Ever smoking |  | -4.8 (-6.8, -2.7) | 7.2 x 10^-06^ |  |  |

rs2282679: Non-effect allele, A, Effect allele, C; rs12785878: G, T; rs10741657: G, A; rs6013897: T, A.
