# Supplementary material for: The Causal Effect of Vitamin D Binding Protein (DBP) Levels on Calcemic and Cardiometabolic Diseases: A Mendelian Randomization Study
Source: PLoS Med. 2014 Oct 28;11(10):e1001751. doi: 10.1371/journal.pmed.1001751 (PMC4211663; doi:10.1371/journal.pmed.1001751)
Supplement: Table S7 — Association of vitamin-D-associated SNPs rs12785878, rs10741657, and rs6013897 with diseases and related traits, from GWAS meta-analyses. (DOCX) [file pmed.1001751.s009.docx]

**Table S7: Association between vitamin D-associated SNPs, rs12785878, rs10741657 and rs6013897, with diseases and related traits, from GWAS meta-analyses.**

| **Consortium** | **Phenotype** | **SNP** | **Effect estimate** | **P-value** | **Sample size**  **(cases/controls)** |
| --- | --- | --- | --- | --- | --- |
| MAGIC | Fasting glucose (mmol/L) | rs12785878 | 0.004 (-0.004, 0.012) | 0.323 | 46,186 |
|  |  | rs10741657 | 0.003 (-0.004, 0.011) | 0.375 | 46,186 |
|  |  | rs6013897 | -0.004 (-0.013, 0.030) | 0.329 | 46,186 |
| MAGIC | Fasting insulin (pmol/L) | rs12785878 | 0.007 (-0.001, 0.006) | 0.091 | 46,186 |
|  |  | rs10741657 | 0.006 (-0.002, 0.007) | 0.139 | 46,186 |
|  |  | rs6013897 | -0.005 (-0.014, 0.032) | 0.270 | 46,186 |
| GIANT | BMI (kg/m^-2^) | rs12785878 | -0.001 (-0.013, 0.012) | 0.94 | 127,611 |
|  |  | rs10741657 | -0.001 (-0.013, 0.010) | 0.84 | 127,611 |
|  |  | rs6013897 | -0.016 (-0.030,-0.002) | 0.026 | 127,573 |
| ICBP | MAP (mmHg) | rs12785878 | 0.029 (-0.098, 0.156) | 0.650 | 29,124 |
|  |  | rs10741657 | -0.019 (-0.136, 0.099) | 0.755 | 28,219 |
|  |  | rs6013897 | -0.187 (-0.328, -0.045) | 0.010 | 29,060 |
| SUNLIGHT | 25OHD | **rs12785878** | **-** | **2.1 x 10^-27^** | **33,996** |
|  |  | **rs10741657** | **-** | **3.3 x 10^-20^** | **33,996** |
|  |  | **rs6013897** | **-** | **6.0 x 10^-10^** | **33,996** |
| GEFOS | Femoral neck BMD (g/cm2) | rs12785878 | -0.005 (-0.023, 0.013) | 0.602 | 32,961 |
|  |  | rs10741657 | 0.001 (-0.015, 0.017) | 0.932 | 32,961 |
|  |  | **rs6013897** | **-0.041 (-0.061, -0.022)** | **3.04 x 10^-5^** | 32,961 |
|  | Lumbar Spine BMD (g/cm2) | rs12785878 | -0.005 (-0.023, 0.013) | 0.5658 | 31,800 |
|  |  | rs10741657 | -0.008 (-0.025, 0.010) | 0.4149 | 31,800 |
|  |  | rs6013897 | -0.011 (-0.032, 0.009) | 0.6107 | 31,800 |
|  |  |  | OR (95%CI) |  |  |
| DIAGRAM | Diabetes | rs12785878 | 0.97 (0.94, 1.01) | 0.140 | 12,171/56,862 |
|  |  | rs10741657 | 0.98 (0.94, 1.01) | 0.230 | 9,580/53,810 |
|  |  | rs6013897 | 1.04 (1.00, 1.08) | 0.057 | 12,171/56,862 |
| METASTROKE | Ischemic stroke | rs12785878 | 1.00 (0.97, 1.04) | 0.873 | 12,389/62,004 |
|  |  | rs10741657 | 1.01 (0.98, 1.05) | 0.448 | 12,389/62,004 |
|  |  | rs6013897 | 1.00 (0.96, 1.04) | 0.949 | 12,389/62,004 |
| CARDIOGRAM | Coronary artery disease | rs12785878 | 1.01 (0.98, 1.04) | 0.528 | 21,369/61,926 |
|  |  | rs10741657 | 1.00 (0.97, 1.03) | 0.900 | 19,739/60,938 |
|  |  | rs6013897 | 0.99 (0.96, 1.02) | 0.596 | 21,840/62,259 |

P-values <0.0042 are bolded; BMI: body-mass-index; BMD: bone mineral density; MAP: Mean arterial pressure; Sample sizes: Maximum sample sizes indicated either in published paper or publically available summary data; rs12785878: Non-effect allele, G, Effect allele, T; rs10741657: G, A; rs6013897: T, A.
